# One Base Deletion (c.2422delT) in the TPO Gene Causes Severe Congenital Hypothyroidism

**DOI:** 10.4274/jcrpe.1404

**Published:** 2014-09-05

**Authors:** Hakan Cangül, Murat Doğan, Yaman Sağlam, Michaela Kendall, Kristien Boelaert, Timothy G Barrett, Eamonn R Maher

**Affiliations:** 1 Bahçeşehir University Faculty of Medicine, Department of Medical Genetics, İstanbul, Turkey; 2 Yüzüncü Yıl University, Faculty of Medicine, Division of Pediatric Endocrinology, Van, Turkey; 3 Medical Park Göztepe Hospital, Centre for Genetic Diagnosis, İstanbul, Turkey; 4 Southampton Faculty Department of Child Health, Division of Clinical and Experimental Sciences, UK; 5 University of Birmingham, School of Clinical and Experimental Medicine, Centre for Rare Diseases and Personalised Medicine, Birmingham, UK; 6 Cambridge University of Clinical Faculty, Academic Department of Medical Genetics, Cambridge, UK

**Keywords:** TPO gene, mutation, genetics, molecular, congenital hypothyroidism, thyroid dyshormonogenesis

## Abstract

**Ob­jec­ti­ve:** Congenital hypothyroidism (CH) is the most common neonatal endocrine disorder and mutations in the TPO gene have been reported to cause CH. Our aim in this study was to determine the genetic basis of CH in two affected individuals coming from a consanguineous family.

**Methods:** Since CH is usually inherited in autosomal recessive manner in consanguineous/multi-case families, we adopted a two-stage strategy of genetic linkage studies and targeted sequencing of the candidate genes. First, we investigated the potential genetic linkage of the family to any known CH locus using microsatellite markers and then screened for mutations in linked-gene by Sanger sequencing.

**Results:** The family showed potential linkage to the TPO gene and we detected a deletion (c.2422delT) in both cases. The mutation segregated with disease status in the family.

**Conclusion:** This study demonstrates that a single base deletion in the carboxyl-terminal coding region of the TPO gene could cause CH and helps to establish a genotype/phenotype correlation associated with the mutation. The study also highlights the importance of molecular genetic studies in the definitive diagnosis and accurate classification of CH.

## INTRODUCTION

Congenital hypothyroidism (CH) is the most common neonatal endocrine disorder with an incidence of 1/3500 live births. It causes mental retardation and growth delay unless a timely and proper treatment is introduced (1). Molecular genetics analyses facilitate definitive diagnosis and accurate classification of CH and might also describe patient-specific targets for alternative treatment of the disease.

About 2% of CH is familial and to date, 11 causative genes have been described for the pathogenesis of inherited CH (2). Table 1 shows the details of all these loci and associated clinical phenotypes. Some of these genes are associated with primary thyroid dysgenesis (3,4) and some with thyroid dyshormonogenesis (TDH) (5). Currently, there are seven genes known to cause congenital TDH which encode for proteins involved in thyroid hormone biosynthesis (6).

Major steps in thyroid hormone synthesis include oxidation and covalent linkage of iodide to tyrosine residues of thyroglobulin (TG) catalysed by thyroid peroxidase (TPO) enzyme (7). This latter reaction requires hydrogen peroxide (H2O2) as the final electron acceptor (8,9). Therefore, the generation of H2O2) is a critical step in the synthesis of thyroid hormones (10). A defect in the system that generates H2O2), resulting in CH, has been reported previously (11,12,13). Two dual oxidases (DUOX1 and 2) have recently been identified as the components of the thyroid H2O2)-generating system (14,15). TPO is a thyroid-specific heme peroxidase localised in the apical membrane of thyrocytes and plays a central role in the thyroid hormone biosynthesis. Mutations in TPO causing permanent CH are mostly inherited in an autosomal recessive fashion and to date, more than 60 distinct mutations have been described in this gene (8,9).

To investigate the genetic background of CH, our group of investigators have developed a two-tier strategy combining genetic linkage studies and full sequencing of candidate genes in familial cases and to date have identified several mutations in different CH genes (16,17,18,19,20,21,22,23,24,25,26,27,28). In the current study, we aimed to determine the genetic cause of CH in a consanguineous family with two affected siblings. Here, we report a homozygous one-nucleotide deletion (c.2422delT) in the TPO gene detected in both cases and its associated clinical phenotypes.

## METHODS

The genetics of CH reported in our previous studies (18,19,20,21,22,23) were investigated in two new cases born to a consanguineous Turkish family. When the older sister was first diagnosed at the age of nine months, she had growth retardation. Her hormone levels before treatment were thyroid stimulating hormone (TSH): 720 µIU/mL (normal range: 0.3-5), total thyroxine (T4) <0.9 µg/dL (normal range: 6.6-17.2), free T4 (fT4) <0.09 ng/dL (normal range: 0.9-2.3), total triiodothyronine (T3) <0.17 ng/mL (normal range: 1.05-3.45) and free T3 (fT3) <0.2 pg/mL (normal range: 2-5). This patient is currently 12 years old and has severe mental retardation. Her younger sister was diagnosed much earlier, on the eighth day of birth and had thyroid enlargement detected by thyroid scintigraphy. Hormone levels in this second patient were TSH: 860 µIU/mL (normal range: 0.3-5), total T4 <0.7 µg/dL (normal range: 6.6-17.2), fT4 <0.12 ng/dL (normal range: 0.9-2.3), total T3 <0.15 ng/mL (normal range: 1.05-3.45) and fT3 <0.2 pg/mL (normal range: 2-5). This patient is now eight years old and her development is normal. Hypothyroid phenotype is permanent in both cases and they require continuous T4 treatment. The parents and a healthy sister are all free of any signs or symptoms of hypothyroidism. Informed consent was obtained from the family and venous blood samples were collected from all family members. All procedures performed were in accordance with the Declaration of Helsinki and the study was approved by relevant IRBs/Ethics Committees. DNA was extracted by using standard methods and stored at -20 °C until analysed.

**Potential Linkage Analysis**

First we performed linkage analysis to all 11 known CH loci in all family members with microsatellite markers (Table 1). Fluorescent labelling of one oligonucleotide of each primer pair enabled the sizing of PCR products in a capillary electrophoresis machine by the use of GeneMapper v4.0 software suite (Applied Biosystems, Warrington, UK). By combining genotypes for each microsatellite marker, we constructed haplotype tables for each family member. As autosomal recessive inheritance was assumed in consanguineous families, homozygosity of a particular haplotype for a locus in cases accompanied by heterozygosity of the same haplotype in both parents was taken as suggestive of linkage to that locus.

**Direct Sequence Analysis of the TPO Gene**

The TPO gene was sequenced by conventional Sanger sequencing and the primer sequences and PCR conditions are available upon request. PCR products were size-checked on 1% horizontal agarose gels and cleaned up using MicroCLEAN (Microzone, Haywards Heath, UK) or gel-extracted using QIAquickTM Gel Extraction kit (Qiagen, Crawley, UK). The purified PCR products were sequenced in both forward and reverse directions using the ABI BigDye Terminator v3.1 Cycle Sequencing kits on an ABI Prism 3730 DNA Analyzer (Applied Biosystems, Warrington, UK). Analysed sequences were then downloaded using Chromas software and assessed for the presence of alterations.

## RESULTS

**Figure 1:**
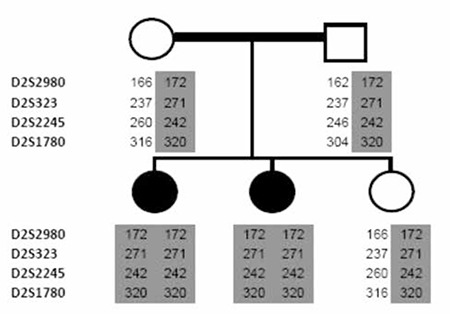
The scores of microsatellite marker analysis surrounding the TPO locus in family members. The markers used are listed on the left and the disease associated haplotype is shaded in grey

Haplotype tables were constructed for each family member by combining the scores for each marker to observe the segregation of the genotype along with the disease status. The linkage analysis using these tables indicated a potential linkage to the TPO locus in the family, i.e. both CH cases were homozygous for a disease-associated haplotype, while both parents and the healthy sister were all heterozygous for the same haplotype (Figure 1). Assuming an autosomal recessive inheritance model which is the most likely pattern in consanguineous families, these results suggested that the disease-associated haplotype segregated with the disease status in the family. Therefore, we proceeded to sequence the coding region (and flanking sequences) of the TPO gene in all members of the family.

**Figure 2:**
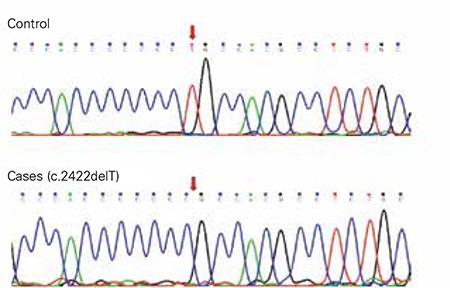
Sequence electropherograms of exon 14 in the TPO gene showing the homozygous deletion c. 2422delT in the cases (bottom panel) compared to the control (upper panel). Red arrows point to the site of the deletion

Direct sequencing analysis revealed a homozygous deletion of T nucleotide in codon 808 of the TPO gene (c.2422delT) in both affected siblings, which results in a frameshift mutation and leads to an early stop codon in exon 14 of the gene (p.Cys808AlafsX24). The parents and the unaffected sister all carried the mutation at heterozygous state which is consistent with the linkage results (Figure 2). Codon 808 is located in exon 14 of the 17-exon TPO gene and this mutation is expected to result in a truncated protein product which will lack carboxy-terminal catalytic domains. This in turn could render TPO protein completely non-functional. 

## DISCUSSION

It is well known that CH, if untreated, may lead to severe developmental delay and genetic defects have been long been implicated in the etiology of the disease. Currently, improving genetic analyses provide a powerful tool to unravel the pathogenesis of the disease and as the number of causative genes grows, the underlying molecular mechanisms become clearer in increasing number of patients. This is especially important for TDH as it is often inherited autosomal recessively where both parents usually are healthy carriers of a mutation in a particular causative gene.

To date, 11 causative genes have been described for CH, seven (TPO, TG, NIS, PDS, IYD, DUOX2, DUOXA2) of which are associated with TDH phenotype and encode proteins involved in the biosynthesis of thyroid hormones (6). We developed a two-tier strategy, combining genetic linkage and sequencing techniques, to investigate the mutations in all causative CH genes, but our efforts were most fruitful with the TDH phenotype. This strategy is still cost-effective compared to full sequencing of all known genes as some of these genes are considerably large. We have recently also been engaged in developing a new testing strategy based on next generation sequencing (NGS) technology covering all causative CH genes in one set. As the prices of NGS are constantly decreasing, in the near future, it might be feasible just to sequence all known-genes in all CH patients. 

In the present study, exploiting the same strategy, we delineated the genetic background of the disease in a consanguineous family and detected a homozygous TPO deletion (c.2422delT) in both affected siblings which segregated with the disease status in the family, i.e. both obligatory carrier parents and one unaffected sibling were all heterozygous for the same mutation. This mutation was first described by Bakker et al (29) as pathogenic and associated with total iodine organification defect. These authors have concluded that this mutation entirely abolishes the function of the TPO enzyme. The severe phenotype in their patients was also evidenced by very low plasma thyroid hormone concentrations (both of T3 and T4) and highly elevated TSH levels. Radioiodine uptake and perchlorate discharge test results for our cases were not available, but the very low thyroid hormone levels associated with very high levels of TSH in our patients are in line with their observations and confirm the severe phenotype caused by c.2422delT mutation. Therefore, it would be plausible to suggest that there is a firm genotype/phenotype relationship associated with this TPO mutation.

In conclusion, we state that in these two patients, the CH was caused by c.2422delT TPO mutation and that this mutation is associated with severe CH. Our study contributes to the establishment of a firm genotype/phenotype relationship associated with this mutation. Molecular genetic studies as such would allow the description of exact etiology and pathogenic mechanism of the disease in particular patients.

**Acknowledgements**

We are grateful to the family members who agreed to participate in this study.

## Figures and Tables

**Table 1:**
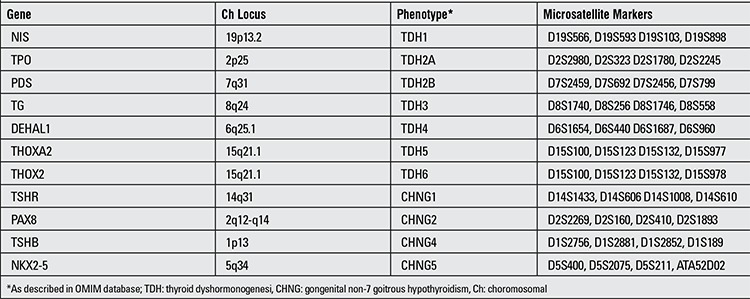
Genes causing congenital hypothyroidism, associated phenotypes and microsatellite markers used for their linkage analysis
